# Spatial congruence in language and species richness but not threat in the world's top linguistic hotspot

**DOI:** 10.1098/rspb.2014.1644

**Published:** 2014-12-07

**Authors:** Samuel T. Turvey, Nathalie Pettorelli

**Affiliations:** Zoological Society of London, Institute of Zoology, Regent's Park, London NW1 4RY, UK

**Keywords:** biocultural diversity, language evolution, New Guinea, threatened languages

## Abstract

Languages share key evolutionary properties with biological species, and global-level spatial congruence in richness and threat is documented between languages and several taxonomic groups. However, there is little understanding of the functional connection between diversification or extinction in languages and species, or the relationship between linguistic and species richness across different spatial scales. New Guinea is the world's most linguistically rich region and contains extremely high biological diversity. We demonstrate significant positive relationships between language and mammal richness in New Guinea across multiple spatial scales, revealing a likely functional relationship over scales at which infra-island diversification may occur. However, correlations are driven by spatial congruence between low levels of language and species richness. Regional biocultural richness may have showed closer congruence before New Guinea's linguistic landscape was altered by Holocene demographic events. In contrast to global studies, we demonstrate a significant negative correlation across New Guinea between areas with high levels of threatened languages and threatened mammals, indicating that landscape-scale threats differ between these groups. Spatial resource prioritization to conserve biodiversity may not benefit threatened languages, and conservation policy must adopt a multi-faceted approach to protect biocultural diversity as a whole.

## 1. Introduction

Similarities between biological evolution and the emergence of new languages have been recognized since the nineteenth century [1,2]. Languages have been described as species whose phenotypes correspond to no genotypes [3], as they share several key evolutionary properties and processes with biological species [4–6]. Studies of language change have therefore drawn heavily upon analytical approaches derived from evolutionary biology [7–10], and linguistic diversity is frequently referred to as a form of biodiversity [11,12].

At continental and global scales, several studies have demonstrated significant spatial overlap between geographical regions with high levels of linguistic richness and high levels of species richness, notably for flowering plants and several vertebrate groups, with high levels of combined ‘biocultural diversity’ in the Neotropics, central Africa, south and southeast Asia and the Pacific region [13–19]. This large-scale positive correlation is likely to reflect a functional connection, possibly resulting from similar spatial processes being responsible for driving diversification in both biological and linguistic evolution such as topographic barriers to dispersal and gene flow [20], latitudinal gradients and climatic variability [13,21,22] or parasite-mediated diversification [12]. Alternatively, increased linguistic richness may be generated by a high level of diversity in biological resources, through processes such as cultural adaptation at finer spatial scales or reduced necessity for wide-scale communication and resource-sharing in more biodiverse environments [19]. Spatial congruence has also been demonstrated at a global scale between geographical regions with high numbers of threatened vertebrate species and threatened languages [17,19]. Although rates of biodiversity decline and language decline over recent decades show different global patterns [23], it is possible that extinction risk in both species and languages may similarly share a functional connection, in this case associated with increasing human population movement, expansion and globalization [17,24,25].

However, it has also been suggested that a unified theory of biocultural diversity is not possible and that different explanations are required to account for relative patterns of language and species richness in different regions [15]. Indeed, languages and species show fundamental evolutionary differences as well as similarities, such as evolutionary rates of different orders of magnitude [26], making it harder to identify causal factors responsible for the development and maintenance of biocultural diversity. In particular, there is still little understanding of the relationship between linguistic and species richness at different spatial scales, and previous studies have stressed the need to investigate relative distributional patterns of languages and species at a range of local and regional levels as well as at a global level [27].

The large tropical island of New Guinea (comprising Indonesia's Papua and West Papua provinces, and the independent country of Papua New Guinea) is the most linguistically complex and diverse region in the world and dominates global patterns and analyses of linguistic richness and diversity [19]. Although it only contains 0.1% of the world's human population and 0.4% of its land area, approximately 1000 of the world's 6900 languages are spoken there, representing an unparalleled level of linguistic diversity comprising over 30 distinct language families and almost as many language isolates [28]. These languages exhibit enormous variation and many unusual properties; for example, the highest diversity of body-part counting systems is found in New Guinea [25]. The region is characterized by village-based subsistence economies dependent upon forest resources, with clans distributed across multiple villages; languages are spoken by an average of only 3000 speakers spread across 10–20 villages, although as many as one-third have fewer than 500 speakers [28]. The existence of numerous small language groups in New Guinea appears to be a long-term stable phenomenon at a state of linguistic equilibrium [24]. However, almost a quarter of the languages spoken across New Guinea are now threatened with extinction [29], primarily as a result of recent changes in external socio-cultural pressures that are also associated with an ongoing regional loss of biodiversity [30].

New Guinea's extremely high levels of linguistic richness have been variously hypothesized to be associated with disaggregation and fragmentation of human populations, as a result of the island's mountainous topography, malaria (at mid/low elevations), or the need to periodically leave land fallow; continuous self-sufficiency of small communities permitted by the island's highly productive tropical ecosystems; and/or the inability of any individual language to become numerically or economically powerful enough to restrain regional linguistic diversification as a result of clan-based social structures rather than state formation [24,28]. Although relative time since settlement may have little effect on modern-day levels of language richness [17,31], it has been suggested that human colonization of the Sahul (New Guinea + Australia) region since around 50 000 BP may have permitted a considerably longer time period for the evolution of high regional levels of language richness than in many other parts of the world [9]. Repeated waves of immigrant populations have further added to the region's linguistic complexity rather than replacing earlier arrivals, with esoterogeny (linguistic character displacement between neighbouring communities) and widespread multilingualism supporting further diversification [28].

Irrespective of the specific mechanism(s) by which New Guinea attained its incredible levels of language richness, focusing on this region as a study area for better understanding patterns and processes of linguistic evolution is an obvious research priority. New Guinea is also an area of extremely high biological diversity and is recognized as a global priority for conservation under international biodiversity frameworks [30,32]. However, the relationships between patterns of linguistic and species richness and threat below the country level have not yet been investigated within the megadiverse New Guinea region, nor indeed within other geographical regions also containing high levels of both linguistic and species richness, despite this being the spatial scale at which the greatest level of diversification in both languages and species has taken place. In the absence of such studies, it is not yet possible to determine the level of functional connection between either diversification or extinction in languages and species, or to characterize the relationship between linguistic evolution, ecology and biogeography.

Based on the results of previous global-scale studies, we may expect spatial patterns of linguistic and biological species richness, and also potentially threat, to be positively correlated across New Guinea's diverse range of landscapes. If there is indeed a close functional connection between either origination and/or extinction in languages and species, either through direct causation or through indirect correlation with other environmental drivers (and these drivers interact in similar ways with both languages and species), we may expect these relationships to be maintained across a series of spatial scales and to show similar trends in response to regional variation in major environmental parameters. However, if environmental or sociological factors other than the observed distribution of species richness and threat play a key role in driving the observed distribution of language richness and threat, then we would expect to detect variation in the strength and pattern of these relationships in response to external factors or at different spatial scales.

In order to test these hypotheses and better understand the spatial structure of biocultural diversity across a single megadiverse region, we investigated patterns of spatial covariation in New Guinea at a series of spatial scales between (i) language richness shown by human populations and (ii) species richness shown by non-human mammals, the vertebrate group representing the closest biological analogue to humans in terms of phylogenetic placement, body size and key ecological attributes, and one of the very few higher taxonomic groupings for which relatively detailed and standardized recent geographical data are available across all species. Our results provide the first quantitative assessment of relative spatial patterns for different components of biocultural diversity in this megadiverse region.

## 2. Material and methods

### (a) Data

A shapefile detailing the spatial distribution of all New Guinean terrestrial mammal species was obtained from the IUCN Global Mammal Assessment [33]. Information on the spatial distributions of New Guinean languages was sourced from [29]; maps from this reference were scanned and geo-referenced to create a shapefile detailing the distribution of languages across New Guinea.

Only data for the main island of New Guinea (land area = 786 000 km^2^) were used for analysis, for two reasons. First, levels of both mammalian and linguistic diversity are relatively low on many individual offshore New Guinean islands [29,33,34], with patterns of island diversity in both species and languages separated by marine barriers likely to be driven by similar processes of allopatric differentiation, whereas the primary purpose of our investigation was to determine the relationship between variation in linguistic and species diversity across more complex contiguous landscapes. Second, most of the New Guinean offshore islands are relatively small, preventing investigation of how changes in spatial scale shape the relationship between biodiversity and language diversity.

Elevation data were obtained from the Advanced Spaceborne Thermal Emission and Reflection Radiometer Global Digital Elevation Model [35]. Mammal species' threat status followed Ref. [36], with species assessed as either Vulnerable (VU), Endangered (EN) or Critically Endangered (CR) all considered threatened with extinction [37]. Language threat status followed the Expanded Graded Intergenerational Disruption Scale [29,38], with languages assessed as either threatened, shifting, moribund, nearly extinct, dormant or extinct since 1950 all considered threatened with extinction.

### (b) Methods

All geo-referenced data were transformed into a Mercator equal area projection to ensure consistency in projections among maps. In order to investigate the effect of spatial scale on the relationship between mammal species richness and language richness, grids of various spatial resolutions (200, 150, 100 and 50 km) were created from the Mercator equal area projection of the study area in ArcGIS 9.3 [39] (electronic supplementary material, figure S1). This range of grid cell sizes was chosen as it provided the best trade-off between sample size (i.e. possible number of grid elements per resolution) and spatial accuracy of available shapefiles. Whereas numerous different mammal species co-occur sympatrically in diverse terrestrial communities, languages instead typically display mutually exclusive allopatric spatial distributions [29]. Across the New Guinea mainland, the mean spatial area occupied by a language is 769.65 km^2^ (range = 0.02–14 542 km^2^, s.d. = 1518.4) and 92.1% of languages have a native spoken range of less than 2500 km^2^, i.e. 50 × 50 km; even grid cells at this highest chosen spatial resolution contain a mean of 5.80 languages per cell (table 1), permitting analysis of the relationship between language and species diversity at all selected spatial resolutions.

**Table 1.** Details of different grid resolutions analysed for the New Guinea mainland, with associated Pearson correlation coefficient (*r*) between linguistic and mammal species richness, and output (*p-*value) of the associated Pearson's product–moment correlation test.

grid resolution (km)	number of mainland cells	languages (mean, range, s.d.)	mammal species (mean, range, s.d.)	*r*	*p-*value
50	363	5.80 (0–35, 4.33)	62.52 (29–113, 23.72)	0.28	<0.001
100	106	13.75 (2–60, 11.41)	70.80 (30–124, 27.06)	0.35	<0.001
150	49	23.31 (4–126, 22.32)	80.31 (34–132, 29.17)	0.43	0.002
200	29	38.34 (7–113, 30.99)	90.17 (34–140, 31.38)	0.53	0.003

Using the Hawth's Analysis toolbox in ArcGIS 9.3, the number of languages, number of mammal species and mean elevation were determined for each grid cell at each spatial resolution. Cells with areas that did not encompass at least 25% of the mainland were not considered for further analysis. Correlations between total levels of language and species richness and elevation, and levels of threatened language and species richness, were assessed using Pearson's product–moment correlation tests. All statistical analyses were performed in the statistical package R [40].

## 3. Results

**Figure 1.**
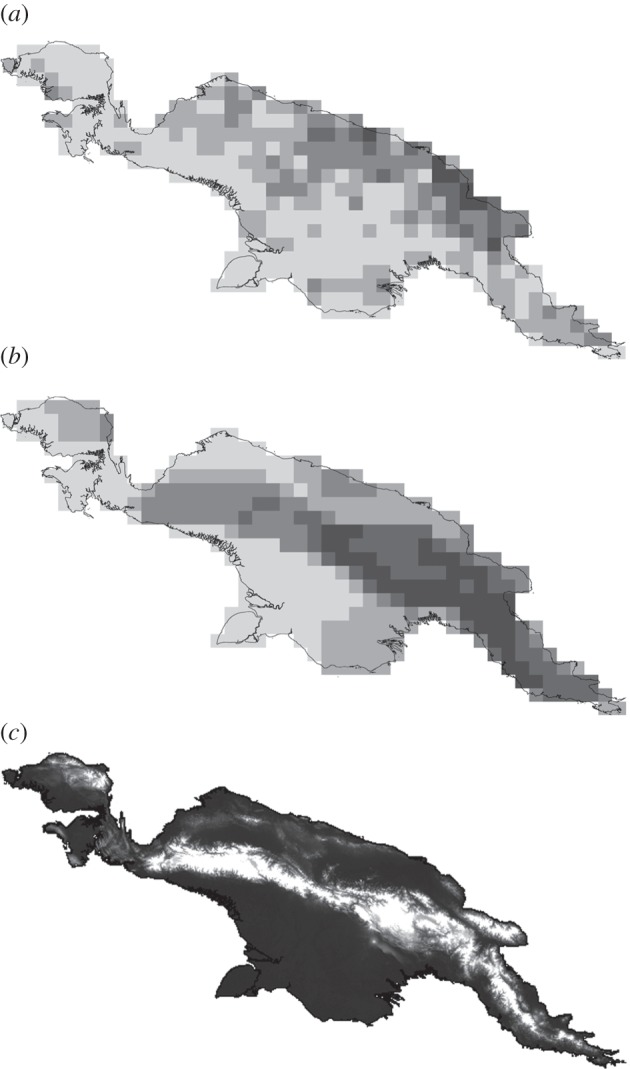
Spatial distributions of (*a*) language richness, (*b*) mammal richness and (*c*) elevation across the New Guinea mainland, at a 50 km grid cell resolution. Five selected richness bins in (*a*,*b*), increasing in richness from paler to darker squares, represent natural breaks in the distribution of respective richness indices (languages: 0–4, 5–7, 8–12, 13–19, 20–35; mammals: 0–45, 46–60, 61–79, 80–96, 97–113).

Data on the spatial distribution of 242 mammal species and 871 languages across the New Guinea mainland were available for analysis. Significant positive relationships between the distribution of language richness and mammal species richness across this region were demonstrated at all four spatial resolutions considered (200, 150, 100 and 50 km) (table 1 and figure 1; electronic supplementary material, figure S1). However, although there is close spatial congruence between areas with high levels of both language richness and mammal species richness in some regions of the New Guinea mainland (e.g. Huon Peninsula), the overall island-wide correlations that we detected are driven by spatial congruence in areas with low levels of both language richness and mammal species richness. Whereas there is statistically strong spatial correlation between language richness and mammal species richness at the highest level of spatial resolution (50 km) for the subset of grid cells containing either lower than median levels of languages (*r* = 0.24, *p* < 0.001) or mammal species (*r* = 0.40, *p* < 0.001), there is no spatial correlation for grid cells containing either higher than median levels of languages (*r* = 0.08, *p* = 0.32) or mammal species (*r* = 0.04, *p* = 0.61) (median languages/cell = 5, median mammal species/cell = 55 at 50 km resolution). Large areas of the south (notably in the southern New Guinea lowlands and Trans-Fly region) and west (Bird's Head Peninsula) of New Guinea display low levels of both language and mammal richness, whereas the highest observed levels of mammal richness are distributed across the Central and Eastern Highlands, and the highest observed levels of language richness are instead distributed closer to the northern coast (figure 1).

**Figure 2.**
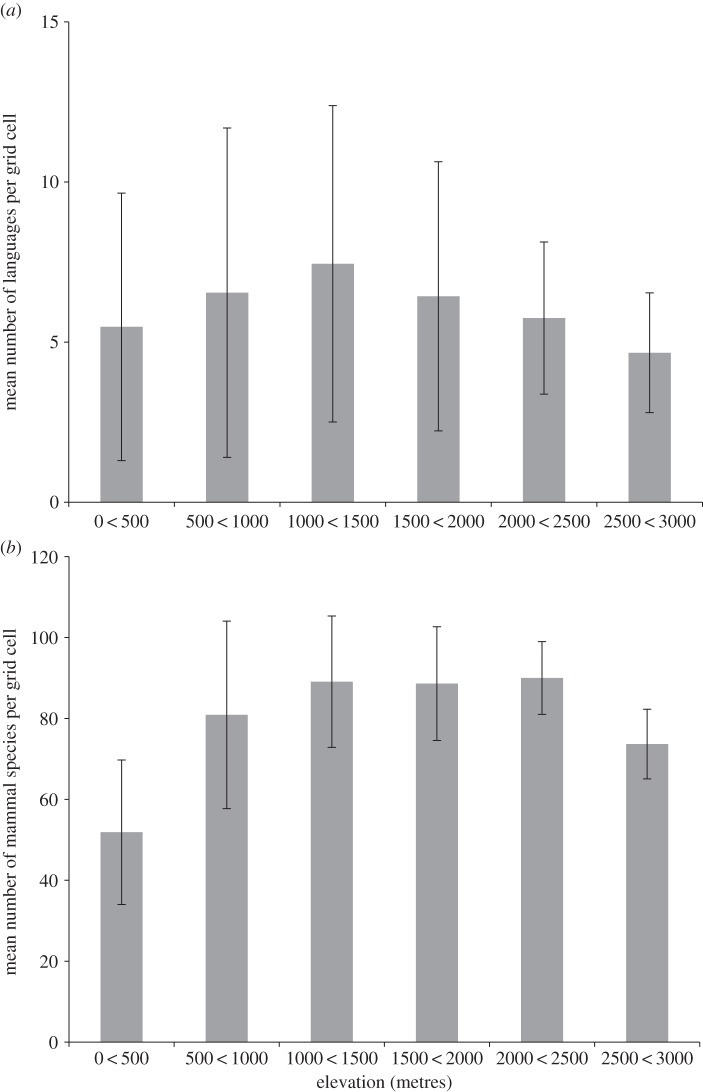
Mean ± 1 s.d. for (*a*) number of languages and (*b*) mammal species per 50 km grid cell at different elevations across the New Guinea mainland.

Land elevation varies greatly across the New Guinea mainland, in places almost reaching 5000 m.a.s.l. (figure 1*c*), although 67.4% of all 50 km grid cells have a mean elevation below 500 m. Topography is a major determinant of New Guinea mammal species richness, with a consistent positive relationship observed between species richness and elevation independent of the spatial resolution considered (table 2). However, lower levels of spatial congruence were detected between topography and language richness; a significant positive relationship between mean elevation and language richness could only be detected at higher levels of spatial resolution, with the significance of the relationship disappearing with grid cells greater than 100 km. A greater proportion of languages than mammals occur at lower elevations across New Guinea (figure 2), and the spatial correlation between language and mammal richness as indexed by Pearson's coefficient decreases from 0.31 below 500 m to 0.20 above 500 m.

**Table 2.** Pearson correlation coefficient between the mean elevation per grid cell and both language richness and mammal species richness, for different spatial resolutions. *p*-values of the associated Pearson's product-moment correlation tests are provided in brackets.

grid resolution (km)	language richness	mammal species richness
50	0.11 (*p* = 0.03)	0.61 (*p* < 0.001)
100	0.20 (*p* = 0.04)	0.67 (*p* < 0.001)
150	0.21 (*p* = 0.15)	0.69 (*p* < 0.001)
200	0.29 (*p* = 0.12)	0.72 (*p* < 0.001)

**Figure 3.**
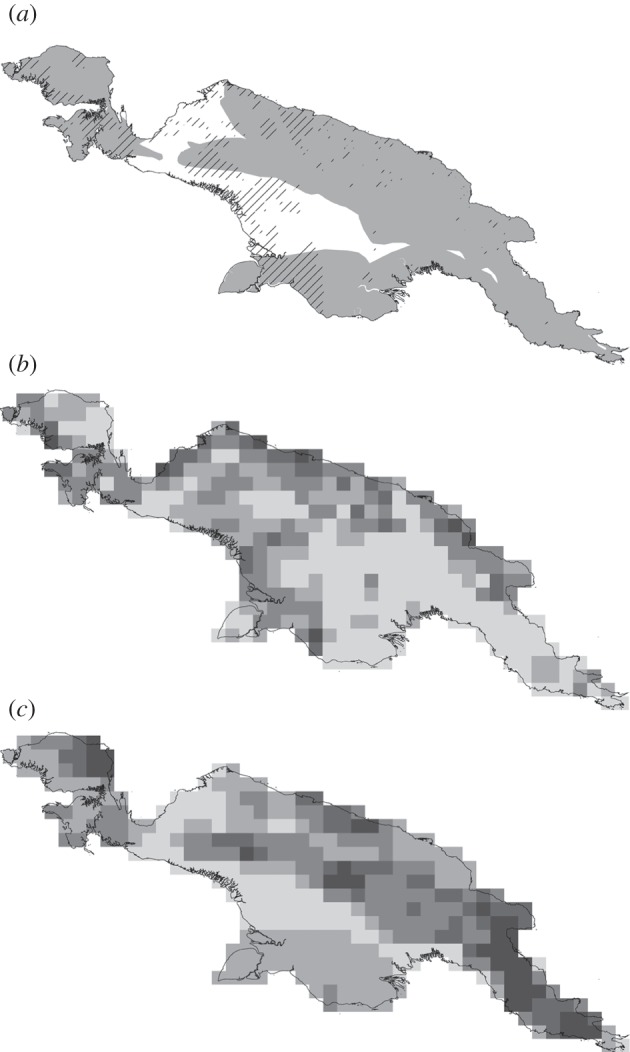
(*a*) Spatial distributions of threatened languages (hashed areas) and threatened mammal species (shaded areas), (*b*) threatened language richness (50 km grid cell resolution) and (*c*) threatened mammal richness (50 km grid cell resolution) across the New Guinea mainland. Five selected richness bins in (*b*,*c*), increasing in richness from paler to darker squares, represent natural breaks in the distribution of respective richness indices (languages: 0, 1, 2–3, 4–5, 6–9; mammals: 0–1, 2, 3–4, 5, 6–9).

There are 34 globally threatened mammal species distributed across the New Guinea mainland (10 Critically Endangered, 11 Endangered and 13 Vulnerable), representing 14.0% of all species in the region [33]; no species have definitely become extinct during recent history, although some may have recently disappeared, e.g. Telefomin cuscus *Phalanger matamin*, New Guinea big-eared bat *Pharotis imogene*. There are 214 globally threatened (including recently extinct) languages distributed across this region (112 threatened, 46 shifting, 27 moribund, 25 nearly extinct, one dormant and three extinct since 1950), representing 24.6% of all languages in the region [29]; three languages that occur in both Papua New Guinea and Indonesia are only threatened on the Indonesian side of the border. Large areas of the New Guinea mainland contain both threatened languages and threatened species (figure 3). However, in direct contrast to the spatial relationship between overall levels of language richness and mammal species richness, there is a statistically significant negative spatial correlation across the New Guinea mainland as a whole between areas with higher levels of threatened languages and areas with higher levels of threatened mammal species when investigated at the 50 km level of spatial resolution (*r* = −0.11, *p* = 0.04).

## 4. Discussion

The significant positive correlation between the distribution of linguistic and mammal species richness across the mainland of New Guinea at all spatial scales considered in our analysis demonstrates the likely existence of a functional relationship between these two components of biocultural diversity at a much finer geographical resolution than recognized in previous studies, although it should be identified that the variation explained by this relationship increases as spatial resolution decreases (table 1). Scale is a well-known determinant of patterns and processes in ecology [41–43], with many fundamental macroecological relationships, such as the nature and strength of the link between species richness and primary productivity, demonstrated to be strongly scale-dependent [44–46]. Cross-taxon congruence in spatial patterns of species richness and related metrics such as β-diversity across different vertebrate groups are also scale-dependent and geographically variable, with lower levels of congruence at finer spatial resolutions [47,48]. However, congruence in overall richness in languages and mammals is now shown to extend from the global and country scales as far as infra-island landscape-level resolution, the spatial level at which allopatric barriers might operate between populations during the speciation process for many groups [49]. This pattern contrasts with demonstrated non-congruence between mammals and other taxonomic groups in New Guinea in indices of infra-island diversity, notably insect communities in lowland rainforests, which display markedly lower levels of β-diversity across New Guinea probably as a result of reduced host specificity or dispersal limitation [50]. We encourage further investigations into the relationship between linguistic diversity and additional components of biodiversity as complete data on the distributions of other major taxonomic groups become available for New Guinea.

These findings permit more meaningful insights to be made into linguistic ecology and the environmental factors responsible for the origination and maintenance of language richness. The positive spatial correlation observed in linguistic and mammal species richness suggests that similar spatial processes may have been responsible for driving diversification at a landscape level in both linguistic and mammalian evolution in New Guinea. Indeed, environmental features such as elevational gradients have previously been proposed as common drivers of diversification and regional endemism in both languages and mammal species in New Guinea [24,28,34,51]. However, the differing observed associations between richness in languages and mammals in relation to topography, and the reduced spatial congruence between areas with high levels of richness in both groups, indicates that the relationship may not be so straightforward. It is possible that languages and mammals have diversified in different ways in response to similar environmental drivers (e.g. topography, ecosystem productivity) that vary across New Guinea's landscapes. Alternatively, it is possible that different factors are responsible for establishing the statistically similar spatial patterns shown by languages and mammals across New Guinea, or that initial patterns of spatial richness that developed in response to similar factors at an island-wide scale have been secondarily modified by historical processes since the origination of regional diversity in either group.

All New Guinea mammal species for which dated phylogenies are available are known to have diverged long before the first human colonists arrived on the island. For example, the major New Guinea anisomyine rodent radiation occurred during the Pliocene 2.5–1.7 Ma [52], and all modern dasyurid species similarly originated earlier than the Pleistocene [53]. Furthermore, the proposed drivers of spatial patterns of regional mammal diversification are typically associated with processes older than the period of human colonization, including Neogene geological accretion of palaeo-terranes with the northern New Guinea mainland (associated with evolution of restricted-area endemics in isolated massifs [54–56]); and Miocene–Pliocene uplift of the Central Cordillera [57], Quaternary uplift of the Huon Peninsula [58] and Pleistocene climatically-driven environmental fluctuations (all associated with diversification in bandicoots and pademelons [59,60]). These geological and climatic drivers of mammal diversification represent contingent historical events that had concluded by the time of first human arrival.

Conversely, major changes in regional language diversity and richness across New Guinea are instead associated with known Holocene events that have limited spatial congruence with earlier drivers of mammalian diversification, and explain the non-significant relationship between language richness and topography shown at broader spatial scales. A quarter of all New Guinean languages today are in the Austronesian language family, which originated in southeast Asia around 6000 years ago and spread south to New Guinea 4000–5000 years ago, representing New Guinea's most recent major linguistic migration [61]. Austronesian speakers settled primarily along the northern coast of the main island of New Guinea, creating the island's primary linguistic division and leading to greatly elevated levels of language richness through subsequent *in situ* diversification in this largely lowland region [28]. An opposite trend in linguistic diversity has occurred in the New Guinea highlands over the past few centuries, where the development of intensive agriculture based on sweet potato cultivation led to large, dense populations and the associated geographical expansion of a relatively small number of languages in the Trans New Guinea language family along the Central Cordillera at the expense of other linguistic diversity; this linguistic expansion event has been largely restricted to high altitudes because sweet potato agriculture is only possible under these environmental conditions, and endemic malaria limits human population growth and aggregation at lower elevations [24,28]. The linguistic landscape of New Guinea would have been at a radically different equilibrium before these two historical events, and earlier patterns of language richness are likely to have had a stronger positive relationship with topography and shown closer spatial congruence with mammal species richness. Future analysis of the spatial relationship between species richness and non-Austronesian language richness across New Guinea may help to test this hypothesis, and we also encourage further investigation into the spatial relationship between different components of biocultural diversity and other potential environmental drivers of this diversity (e.g. ecosystem productivity).

The significant negative spatial correlation observed across New Guinea between threatened language and threatened mammal species richness contrasts not only with the positive spatial relationship seen in overall levels of language and mammal species richness across the same region, but also with previous studies that have detected positive spatial relationships between threatened linguistic diversity and biodiversity at a global scale [17,19]. Whereas linguistic diversity and biodiversity are globally threatened by comparable broad-scale anthropogenic processes, and some pressures may threaten both integrity of indigenous cultures and their interactions with local wildlife in New Guinea (e.g. social changes leading to breakdown of traditional taboos and associated cultural attitudes towards the environment [62,63]), our results suggest that other specific threats are instead likely to differ between languages and mammals, and may be further influenced by spatial scale and landscape-level variation. Threatened mammal richness is mainly distributed within the New Guinea Highlands (figures 1 and 3), reflecting either the positive relationship observed between overall species richness and elevation or hunting pressure from the larger indigenous human populations that occur at higher elevations on the island; whereas most of New Guinea remains forested [64] and deforestation is primarily affecting lowland regions that contain relatively low mammal species richness [65], New Guinean mammals are likely to be particularly threatened by unsustainable overexploitation [66]. Conversely, threatened language richness is mainly distributed in Indonesian New Guinea at both high and low altitudes, suggesting that documented external socio-political pressures in this region are causing greater disruption to indigenous cultural and linguistic continuity in comparison to the situation in autonomous Papua New Guinea [67].

Our analysis of the spatial patterns of linguistic diversity and biodiversity in the world's most significant language hotspot represents a key step towards a future understanding of the processes that generate and influence regional biocultural diversity, and in particular whether the observed relationship between language richness and species richness represents direct causation or indirect correlation with other environmental drivers. The lack of spatial congruence between the distribution of threat in mammals and languages in New Guinea also demonstrates that spatial prioritization of resources to conserve threatened linguistic diversity at the landscape scale may not provide a comparable degree of benefit to threatened biodiversity, and vice versa. Despite the close spatial relationship between overall linguistic and mammal species richness across a range of spatial scales, global conservation policy will have to adopt a multi-faceted approach to protect both the biosphere and the logosphere.

## Supplementary Material

Figure S1
