# Supplementary figures and images for: Spatial congruence in language and species richness but not threat in the world's top linguistic hotspot
Source: Proc Biol Sci. 2014 Dec 7;281(1796):20141644. doi: 10.1098/rspb.2014.1644 (PMC4213640; doi:10.1098/rspb.2014.1644)

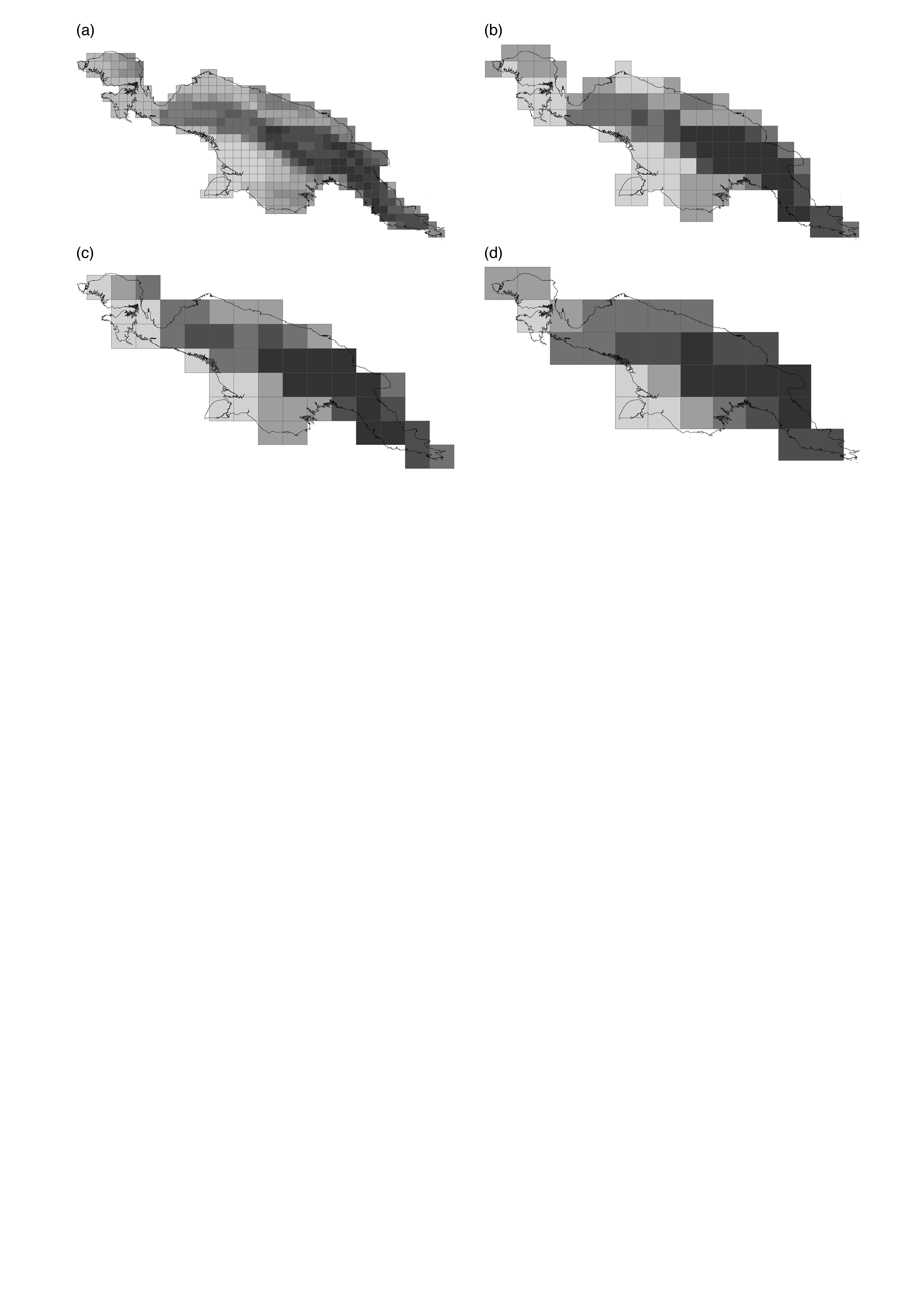

Supplement: Figure S1 [file rspb20141644supp1.tif]
